# Excess body mass index loss predicts metabolic syndrome remission after gastric bypass

**DOI:** 10.1186/1758-5996-6-1

**Published:** 2014-01-02

**Authors:** Mário Nora, Marta Guimarães, Rui Almeida, Paulo Martins, Gil Gonçalves, Mariana Santos, Tiago Morais, Cláudia Freitas, Mariana P Monteiro

**Affiliations:** 1 Department of General Surgery, Centro Hospitalar de Entre o Douro e Vouga, Santa Maria da Feira, Portugal; 2 Endocrine Unit, Centro Hospitalar de Entre o Douro e Vouga, Santa Maria da Feira, Portugal; 3 Department of Anatomy, Multidisciplinary Unit for Biomedical Research (UMIB), ICBAS, University of Porto, Rua Jorge Viterbo Ferreira, 228, PORTO 4050-313, Portugal

**Keywords:** Bariatric surgery, Gastric bypass, Metabolic syndrome, Obesity

## Abstract

**Background:**

Metabolic syndrome (MS) is a condition associated with obesity that identifies individuals with increased cardiovascular risk. Gastric bypass improves several MS components, such as glucose, lipid metabolism and hypertension. The aim of this study was to evaluate the effect of long-limb gastric bypass on the remission of MS criteria associated with morbid obesity.

**Methods:**

Obese patients who met the “harmonized” criteria for MS (n = 153) that underwent laparoscopic Roux-en-Y gastric bypass (LRYGB) with a long biliopancreatic limb were prospectively evaluated with regards to body weight, body mass index (BMI), percentage of excess BMI lost (% EBMIL), fasting glucose, blood pressure and lipid profile up to 36 months after surgery.

**Results:**

Before surgery, patients had a BMI of 44.3 ± 0.5 kg/m^2^; 66% were under anti-diabetic treatment; 78.4% were under anti-hypertensive treatment and 44.3% were under anti-dyslipidemic treatment. After a mean follow-up time of 2.4 ± 0.1 years, MS remission rates were 32.7% at 6 months, 69.7% at 12 months, 63.4% at 24 months, and 59.2% at 36 months; when only 32.9%, 43.4% and 15.8% of patients were still under anti-diabetic, anti-hypertensive and anti-dyslipidemic treatment, respectively. The %EBMIL and BMI were the parameters that showed the highest accuracy to predict the MS remission at all-time points after the surgery.

**Conclusions:**

Long limb gastric bypass in obese patients results in significant and sustained weight loss which predicts a high remission rate of MS and allows the discontinuation of drug therapy for several metabolic disturbances in most patients.

## Introduction

Obesity has assumed alarming worldwide proportions in the last decades [1,2]. The number of overweight and obese persons has been estimated to encompass 1.7 billion individuals [3]. Obesity and related co-morbidities affect both patients quality of life and life expectancy [4]. The rise in the rate of obesity has been associated with an increased incidence of many obesity-related co-morbidities, including type 2 diabetes mellitus (T2DM), lipid disorders and hypertension, which are major causes of cardiovascular diseases [5-8]. Obesity and overweight are responsible for the increased risk of mortality due to cardiovascular disease, particularly in the presence of visceral adiposity, a key component in the development of insulin resistance [9] and metabolic syndrome (MS) that has become an important public health concern [10,11].

The first descriptions of the association between T2DM, hypertension and dyslipidemia dates from 1920; but it was Reaven in 1988, who suggested for the first time that these factors tended to occur in the same individual under the form of a syndrome “X”, which included five components: resistance to glucose uptake mediated by insulin, glucose intolerance, hyperinsulinemia, elevated triglycerides, decreased HDL cholesterol and hypertension; all five components were associated with an increased risk of coronary heart disease [12]. In 1998, a WHO advisory group proposed the designation and suggested the first working criteria for MS [13], while later other definitions have been proposed by NCEP- ATPIII (National Cholesterol Education Program - Adult Treatment Panel III, 2001), IDF (International Diabetes Federation, 2005) and more recently the “Harmonized” Criteria [14].

The prevalence of MS varies with age and sex, studied population and criteria used for its definition. The prevalence of MS in the U.S. varies; between 40–59 years is 40% in men and 34% women, while aging is accompanied by a 20% increase in the prevalence of MS [11,15,16]. In high-risk populations, such as relatives of diabetic patients the prevalence of MS increases to 50%; in diabetic patients to 80% and individuals in with impaired glucose tolerance to 40% [15]. In Europe, the reported prevalence of MS is 9.5% in men and 8.9% in women, based on the DECODE study, [17]; while 24% of the adult population in the northern region of Portugal meet the ATPIII criteria for MS [18].

Insulin resistance is considered the central abnormally of MS and T2DM, preceding the disease diagnosis and clinical findings in 5 to 6 years; MS increases the lifetime risk of developing T2DM and is associated with a higher prevalence of cardiovascular disease and cardiovascular death [19], which justify the main interest in identifying patients with MS [20].

At the present time there is no single medication that targets MS. Lifestyle modifications with the aim of reducing the underlying causes: central obesity/overweight and insulin resistance are the cornerstones in the management of MS [21,22]. Aggressive treatment with drug combination therapy targeted to each of the components of the MS is highly recommended as the benefits are well-known in the control on morbidity and mortality [23].

Since most dietary interventions do not achieve more than 10-15% weight loss that is often regained, the medical treatment of obesity and MS rarely allows long lasting or sustained weight loss and MS remission. Bariatric surgery, as a treatment option for obese individuals, has proven to be effective in treating the different components of the MS, as well inducing a significant reduction in the prevalence of MS [22,24,25].

After having demonstrated that LRYGB with a 200 cm biliopancreatic limb in obese patients was associated with a high remission rate of diabetes and improvement of the metabolic control [26], the purpose of the current study was to evaluate the efficacy and safety of the same surgical technique when applied for the treatment of obese patients with metabolic syndrome.

## Methods

### Patients and methods

Obese individuals were selected from the prospective database of patients referred for bariatric surgery at the Department of General Surgery of Centro Hospitalar de Entre o Douro e Vouga (CHEDV), Portugal. The database included 696 patients that underwent laparoscopic gastric bypass, of which 153 patients met diagnostic criteria for MS and were submitted to LRYGB for the primary treatment of obesity.

The inclusion criteria were BMI > 35 kg/m^2^ with diagnostic criteria of MS according to the “harmonized” criteria, consisting in the presence of three abnormal findings out of the following five: abdominal obesity ≥ 102 cm (men) or ≥ 88 cm (women) caucasians; hypertension ≥ 130/85 mmHg; high triglycerides ≥ 150 mg/dl; low HDL <40 mg/dl (men) or <50 mg/dl (women); fasting glucose ≥ 100 mg/dl, or drug treatment for any of these conditions [14]. For abdominal obesity, since the recommended cut points equate to a body mass index of approximately 30 kg/m^2^ according to the definitions of abdominal obesity found in National Institutes of Health obesity guidelines, and as the location of the waist is often difficult to determine rendering the measure less accurate, a BMI > 30 Kg/m^2^ was used as a surrogate marker for visceral obesity [14,21].

Every patient with MS criteria proposed for bariatric surgery for the treatment of obesity during this period of time was enrolled in the study and submitted to LRYGB with a bilio-pancreatic limb of 200 cm as previously described [26]. Subjects were informed of potential risks associated with the surgery and signed an informed consent document, which was approved by the (CHEDV) Institutional Ethical Review Board.

The parameters evaluated in patient follow-up were body weight, BMI, percentage of excess BMI lost (% EBMIL), fasting blood glucose, blood pressure and lipid profile up to 36 months after surgery and ongoing medical treatment before and after surgery. The patients were assessed for the presentation of MS parameters at each time point of the study and no time limit has been defined for normalization of the metabolic parameters. Patients were classified as being in remission if no longer had diagnostic criteria of MS.

### Statistical analysis

Results are presented as mean ± standard error of the mean (Mean ± SEM) unless otherwise specified. Comparisons between groups were performed with the Kruskal–Wallis test (one-way ANOVA) followed by the Dunn post hoc test. Ordinal and nominal data were compared using a Chi square test and s p <0.05 was considered significant. To test the accuracy of all parameters in predicting the presence of MS was evaluated using the area under the receiver operating characteristic (ROC) curve. Based on the area under the curve (AUC), the test is considered excellent when the AUC is 0.90 to 1.00; good from 0.80 to 0.90; fair from 0.70 to 0.80; poor from 0.60 to 0.70 and fail if below 0.60. Data was analyzed using the IBM SPSS Statistics 21 and Graphpad Prism 5.04 programs.

## Results

Patients enrolled in the study (n = 153) included 84.3% of females (n = 129) and 15.7% males (n = 24) with a mean age of 48.5 ± 0.7 years and a mean BMI of 44.3 ± 0.5 kg/m^2^ at the time of the surgery. All patients met at least three diagnostic criteria for MS; thus, 66.0% (n = 101) were under anti-diabetic drugs, 78.4% (n = 120) were treated for hypertension and 44.3% (n = 66) were treated for dyslipidemia. The mean follow-up time after surgery was 2.4 ± 0.1 years, and every patient included in the statistical analysis had a minimum follow-up time of 6 months (Table 1).

**Table 1:** Patient demographics and length of follow-up

**Patient characteristics at baseline**	**Number**
**Age (years)**	48.5 ± 0.7
**Male/Female**	24.0:129.0 (15.7%:84.3%)
**Body Weight (kg)**	113.9 ± 1.4
**BMI (Kg/m**^**2**^**)**	44.3 ± 0.5
**Under anti-diabetic treatment**	101.0 (66.0%)
**Under anti-hypertensive treatment**	120.0 (78.4%)
**Under anti-dyslipidemic treatment**	66.0 (44.3%)
**Mean follow-up time (years)**	2.4 ± 0.1

After the surgery, there was a significant reduction in BMI and increase in the percentage excess of BMI lost from 6 months onwards. The BMI was reduced to 32.7 ±0.4 kg/m^2^ at 6 months, reaching its maximum at 12 months (30.8 ±0.4 kg/m^2^), and was followed by a non-significant increase to 31.2 ± 0.5 kg/m^2^ and 32.2 ± 0.7 kg/m^2^ at 24 and 36 months, respectively. The percentage excess of BMI lost increased after the surgery from 63.5 ± 1.5% at 6 months, to 73.4 ± 1.7% at 12 months, followed by a non-significant decrease to 71.6 ± 1.8% at 24 months and 67.8 ± 2.4% at 36 months of follow-up. There was also a significant improvement of all analytical parameters used in the diagnosis of MS, namely a decrease of fasting glucose, systolic and diastolic blood pressure, and triglycerides, as well as an increase in HDL-cholesterol levels. Along with weight loss and improvement of metabolic profiles, the need of medical treatment for those conditions decreased while the discontinuation of drug use increased gradually over time, reaching maximum levels at 24 months of follow-up. The discontinuation rate of the use drugs at 36 months after surgery was 51.2% for anti-diabetics, 44.6% for anti-hypertensives and 64.3% for anti-dyslipidaemic drugs (Table 2).

**Table 2:** Metabolic syndrome remission rate and metabolic profile

**Follow-up time (months)**	**0**	**6**	**12**	**24**	**36**	**P**
**Patients**	n = 153 (100%)	n = 153 (100%)	n = 142 (93%)	n = 112 (73%)	n = 76 (50%)	n.a
**BMI (kg/m**^**2**^**)**	44.3 ± 0.5	32.7 ± 0.4^***(a)^	30.8 ± 0.4^***(a)^	31.2 ± 0.5^***(a)^	32.2 ± 0.7^***(a)^	< 0.001
**EBMIL (%)**	-	63.5 ± 1.5^***(a)^	73.4 ± 1.7^***/**(a/b)^	71.6 ± 1.8^***/*(a/b)^	67.8 ± 2.5^***(a)^	< 0.001
**Patients with MS**	153	103	43	41	31	n.a
**MS remission rate (%)**	0.0	32.7^***(a)^	69.7^***/***(a/b)^	63.4%^***/***(a/b)^	59.2%^***/***(a/b)^	^(a),(b)^p < 0.001
**Fasting glycemia (mg/dL)**	136.4 ± 4.8	95.0 ± 2.4^***(a)^	90.4 ± 1.1^***(a)^	93.7 ± 1.6^***(a)^	93.8 ± 1.9^***(a)^	< 0.001
**Systolic blood pressure (mm Hg)**	147.2 ± 1.5	140.4 ± 1.7^*(a)^	138.3 ± 1.5^***(a)^	139.0 ± 1.7^**(a)^	143.9 ± 2.0	< 0.001
**Diastolic blood pressure (mm Hg)**	88.4 ± 1.0	82.5 ± 1.0^***(a)^	81.6 ± 1.0^***(a)^	81.9 ± 1.0^***(a)^	82.7 ± 1.4^***(a)^	< 0.001
**HDL (mg/dL)**	45.5 ± 0.8	44.8 ± 1.0	52.0 ± 0.9^***/***(a/b)^	56.5 ± 1.0^***/***/*(a/b/c)^	56.4 ± 1.6^***/***(a/b)^	< 0.001
**Triglycerides (mg/dL)**	171.7 ± 7.4	104.0 ± 3.2^***(a)^	94.5 ± 3.3^***(a)^	100.7 ± 5.0^***(a)^	104.6 ± 5.4^***(a)^	< 0.001
**Anti-diabetic treatment**	101 (66.0%)	33 (22.8%)^***(a)^	28 (19.9%)^***(a)^	32 (28.6%)^***(a)^	25 (32.9%)^***/*(a/c)^	^(a)^p < 0.001
						^(c)^p = 0.046
**Anti-hypertensive treatment**	120 (78.4%)	59 (40.7%)^***(a)^	50 (35.7%)^***(a)^	41 (36.9%)^***(a)^	33 (43.4%)^***(a)^	< 0.001
**Anti-dyslipidemic treatment**	66 (44.3%)	13 (9.0%)^***(a)^	13 (9.4%)^***(a)^	12 (10.8%)^***(a)^	12 (15.8%)^***(a)^	< 0.001

*p < 0.05; **p < 0.01; ***p < 0.001; (a) vs baseline; (b) vs 6 months; (c) vs 1 year. Kruskal–Wallis test (one-way ANOVA) with Dunn post hoc test for scale data and Chi Square test for ordinal data.

The number of patients that met diagnostic criteria for MS decreased gradually and significantly after the gastric bypass surgery, the remission rate of MS was 32.7% (n = 103) at 6 months, 69.7% (n = 43) at 12 months, followed by a non-significant increase to 63.4% (n = 41) at 24 months and to 59.2% (n = 31) at 36 months after surgery (Table 2). The prevalence of MS in our population of patients submitted to bariatric surgery was 22% before surgery and decreased to 4.4% 36 months after gastric bypass.

The parameters that showed the highest accuracy to predict the MS remission in all time points (6 months, 1 year, 2 years and 3 years) were the %EBMIL and the BMI, with AUC between 0.75-0.85, which denotes that these parameters are either fair or good in predicting MS. The HDL levels also showed a good accuracy (AUC = 0.80) in predicting MS remission but only in the second year after surgery. The cut-off points of BMI that better defined the resolution of MS were: <30.92 kg/m^2^; <29.74 kg/m^2^; <29.92 kg/m^2^ and <31.24 kg/m^2^, for 6 months, 1 year, 2 years and 3 years follow-up, respectively, or <30.02 kg/m^2^ when considering all time points. The cut-off points of EBMIL that better defined the resolution of MS were: > 68.28%; > 76.89%; > 75.45% and > 69.89%, for 6 months, 1 year, 2 years and 3 years follow-up, respectively, or >66.08% considering all time points. The cut-off point of HDL that better defined the resolution of MS was > 58.50 mg/dL at 2 years after the surgery.

The morbidity associated with the surgical procedure included a major early complications rate of 12.4% (n = 14), which included gastrojejunal anastomosis fistulas 2.6% (n = 4), gastrojejunal anastomosis leaks 5.8% (n = 9), hypovolemic shock 0.65% (n = 1), small bowel perforation 0.65% (n = 1), intra-abdominal abscess 1.9% (n = 3), and perforation of the abdominal esophagus 0.65% (n = 1); and major late complications rate of 1.9% (n = 3). Late minor complications rate was of 35% (n = 53) and included mostly anemia due to iron, folate or vitamin B12 deficiencies that has arose in spite of systematic prescription of broad-spectrum vitamin supplementation. The rate of surgical re-intervention was of 4.5% (n = 7) and the 30 days the mortality rate was of 0.65% (n = 1) (Table 3).

**Table 3:** Morbidity, surgical re-intervention and mortality rate of gastric bypass

**Complication**	**Number of patients**	**%**
**Early major complications**		
**Gastrojejunal anastomosis fístula**	4	2.6
**Gastrojejunal anastomosis leak**	9	5.8
**Hypovolemic shock**	1	0.7
**Small bowel perforation**	1	0.7
**Intra-abdominal abcess**	3	1.9
**Perforation of the abdominal esophagus**	1	0.7
***TOTAL***		12.4
**Late major complications**		
**Anatomotic stenosis**	2	1.3
**Vitamin B1deficiency**	1	0.7
***TOTAL***		2
**Late minor complications**		
**Anemia**	54	35
**Iron deficiency**	23	15
**Vitamin B12deficiency**	52	33
**Folate deficiency**	12	7.8
**Rate of surgical re-intervention**	7	4.5
**30 days mortality**	1	0.7

## Discussion

Weight loss is the primary goal in the treatment of MS, as lifestyle intervention with a modest weight loss has showed to reduce the prevalence and the incidence of MS [27,28].

In our study, patients submitted to LRYGB experienced a significant reduction in BMI and increase in %EBMIL from 6 months after the surgery onwards. The %EBMIL after the surgery increased from 6 months to 12 months, which was followed by a non-significant decrease at 24 months and at 36 months of follow-up, but remaining well above 60%. After the surgery there was a significant improvement in all parameters of the MS “harmonized” criteria, as well as a large discontinuation rate of anti-diabetic, anti-hypertensive and anti-dyslipidemic drug use. The maximum remission rate of MS occurred at 12 months, just as the %EBMIL. Although the maximum remission rate of some individual components of the MS occurred at 36 months, namely T2DM, the same was not found in relation to MS criteria. Despite the increase in length of the biliopancreatic limb, the rates of nutritional deficits were lower than those reported by other series which have used the conventional bypass technique, since the length of the common channel in our technical modification is shorter or not different from other studies, which is the major determinant of malabsorption with a higher potential to impact over the nutritional status [29,30].

Surgical therapy is currently the only treatment for severe obesity that has proved to be effective in the long-term [5]. Bariatric surgery has been demonstrated to be safe and induce the remission of co-morbidities associated with obesity beyond weight reduction [7]. LRYGB has demonstrated to reduce the risk of developing T2DM by approximately 75% [31], induce euglycemia in 83% of patients with impaired fasting glucose or T2DM with a higher probability in patients with the shortest duration and mildest form of T2DM [32], reduce blood pressure, mostly in patients with a shorter preoperative duration of the co-morbid condition [33] and improve abnormal lipid levels [34].

In a previous study from our group, T2DM patients submitted to LRYGB with a long biliopancreatic limb showed high remission rates the disease of 87.9% at 6 months, 92.6% at 12 months and 100% at 36 month of follow-up and an improvement of metabolic control in those that remained diabetic [26]. Pinheiro et al., had also reported an improved resolution of diabetes despite similar weight loss, in super-obese patients submitted to LRYGB with a biliopancreatic limb of 100 cm, compared to the gastric bypass with 50 cm biliopancreatic limb [35], suggesting metabolic benefits of this surgical modification.

Since the improvement of the different components of metabolic syndrome after weight loss has been widely reported, bariatric surgery should be considered an alternative therapy for MS patients with BMIs above 35 kg/m^2 ^[31,36,37], and possibly emerging treatment for patients with BMIs below 35 kg/m^2 ^[38].

The prevalence and remission of MS before and after bariatric surgery, has been described before, however, most studies included small patient samples, had a short follow-up time after surgery, there was no uniformity with regards to the definition MS used and no variable was found to predict resolution of MS. Therefore, the remission rate of MS varies with the criteria used for diagnosis and consequently it is difficult to compare the results between different studies [22,24,39-41].

The prevalence of MS has been shown to decrease significantly from 83.2% to 98.4% after surgery [22,24,39,40]. In a large controlled study from the Mayo Clinic with a mean follow-up time of 40 months that included 180 patients submitted to LRYGB and 157 patients enrolled in a medical weight-reduction program, showed that prevalence of MS decreased from 87% to 29% in the bariatric surgery group and from 85% to 75% in the non-surgical group, as defined by the American Heart Association [21].

In the only long term study of the evolution of MS after different bariatric surgery techniques- vertical banded gastroplasty, LRYGB and biliopancreatic diversion- the authors showed that at 12 months after surgery all procedures were responsible for a similar remission rates of MS, using the IDF classification. However, when revaluated at 7 years of follow-up, the percentage of patients submitted to vertical banded gastroplasty and LRYGB that presented MS resumed to near preoperative values owing to weight regain, which did not occur after biliopancreatic diversion yet at the expense of a higher morbidity rate [42].

Most previous reports of MS remission after bariatric surgery have used the WHO or the NCEP criteria, which do not include pharmacological therapy as MS criteria, therefore the use of the IDF and “harmonized” definitions of the metabolic syndrome lead to a higher prevalence of the metabolic syndrome [10], which could account for the higher prevalence of MS found in our post-surgical patients. To our knowledge, no previous study on bariatric patients has used the most updated “harmonized” diagnosis criteria for MS.

The proposed mechanisms that could explain the efficacy of bariatric surgery in the reversibility of MS include weight loss, the decrease in food intake, reduction of abdominal obesity and insulin resistance, reduction in fatty acid turnover, amelioration of the inflammatory response and improvement of endothelial function [1,43,44]. Changes in incretin hormones after the surgical procedures may have a role that still requires being further investigated and could account specifically for the better results achieved after gastric bypass compared to restrictive procedures in diabetes remission. Our data showed that the %EBMIL and BMI were the best parameters for predicting MS remission, suggesting that in contrast to what is observed regarding diabetes, weight loss is a major determinant of MS remission in the clinical setting, as supported by previous studies [21]. The %EBMIL has been shown to be the best parameter to predict MS improvement in obese subjects after Roux-en-Y gastric bypass [45], while the resolution of MS was demonstrated to be independent of preoperative BMI [46].

## Conclusion

MS associated with obesity is reversible by weight loss attained through bariatric surgery. Long limb LRYGB in obese patients is a safe procedure that results in significant and sustained %EBMIL, which predicts a high remission rate of MS and allows discontinuation of drug therapy for associated metabolic disturbances.

## Competing interests

Dr. Nora is Consultant and Trainer in the Bariatric Advisory Board for Ethicon Endosurgery.

## Authors’ contributions

MN and MG analyzed the data and wrote the manuscript; RA, PM, GG, MS, CF, AB observed the patients, gathered the data, participated in the sequence alignment and draft of the manuscript; TM was responsible for the statistical analysis of the data and MPM designed the study, analyzed the data and edited the manuscript. All authors read and approved the final manuscript.
